# InAsSb Photodiode Fibre Optic Thermometry for High-Speed, near-Ambient Temperature Measurements

**DOI:** 10.3390/s23239514

**Published:** 2023-11-30

**Authors:** Emilios Leonidas, Matthew J. Hobbs, Sabino Ayvar-Soberanis, Hatim Laalej, Callum Fisk, Stephen Fitzpatrick, Jon R. Willmott

**Affiliations:** 1 Department of Material Science & Engineering, University of Sheffield, Sheffield S1 3JD, UK; eleonidas1@sheffield.ac.uk; 2 Sensor Systems Group, Department of Electrical & Electronic Engineering, University of Sheffield, Sheffield S1 4ET, UK; m.hobbs@sheffield.ac.uk (M.J.H.); cfisk1@sheffield.ac.uk (C.F.); 3 Advanced Manufacturing Research Centre (AMRC), Machining Research, Process Modelling & Control Group Centre, Factory of the Future, Rotherham S60 5TZ, UK; s.ayvar@amrc.co.uk (S.A.-S.); h.laalej@amrc.co.uk (H.L.); 4 Advanced Forming Research Centre (AFRC), Paisley PA4 9LJ, UK; s.fitzpatrick@strath.ac.uk

**Keywords:** radiation thermometry, pyrometer, InAsSb, photodiode, infrared radiation thermometer, temperature, measurement, monitoring

## Abstract

Infrared radiation thermometers (IRTs) overcome many of the limitations of thermocouples, particularly responsiveness and calibration drift. The main challenge with radiation thermometry is the fast and reliable measurement of temperatures close to room temperature. A new IRT which is sensitive to wavelengths between 3 μm and 11 μm was developed and tested in a laboratory setting. It is based on an uncooled indium arsenide antimony (InAsSb) photodiode, a transimpedance amplifier, and a silver halogenide fibre optic cable transmissive in the mid- to long-wave infrared region. The prototype IRT was capable of measuring temperatures between 35 °C and 100 °C at an integration time of 5 ms and a temperature range between 40 °C and 100 °C at an integration time of 1 ms, with a root mean square (RMS) noise level of less than 0.5 °C. The thermometer was calibrated against Planck’s law using a five-point calibration, leading to a measurement uncertainty within ±1.5 °C over the aforementioned temperature range. The thermometer was tested against a thermocouple during drilling operations of polyether ether ketone (PEEK) plastic to measure the temperature of the drill bit during the material removal process. Future versions of the thermometer are intended to be used as a thermocouple replacement in high-speed, near-ambient temperature measurement applications, such as electric motor condition monitoring; battery protection; and machining of polymers and composite materials, such as carbon-fibre-reinforced plastic (CFRP).

## 1. Introduction

The measurement of temperature is critical in many sectors of industry and science [1,2,3,4,5,6,7]. The ability to perform fast, accurate, and reliable temperature measurements is essential for understanding temperature-dependent phenomena and improving process efficiency and quality [5,8].

Contact-based thermocouples have been the standard thermometer used for many industrial and scientific measurements because of their low capital and installation costs and their capability of measuring a wide range of temperatures [9]. However, thermocouple-based techniques inherently have limitations, such as requiring precise and invasive contact; low responsiveness and sensitivity, making them ineffective and unreliable for high-speed measurements; low chemical resistance, which can deteriorate measurement accuracy and contaminate the measurand; high rates of calibration drift and hysteresis; and accuracy limitations [5,6,7,8,9,10].

Non-contact techniques, notably infrared radiation thermometers (IRTs), often described as pyrometers in the literature, can overcome many of the limitations of thermocouple techniques, particularly those associated with intimate and invasive contact that could contaminate the measurand [7]. They are capable of high-speed temperature measurements due to their thermal radiation modus operandi, as opposed to the slower thermal conduction process of thermocouples; however, a direct line of sight is required, making them unsuitable for use in harsh and chemically aggressive environments that obscure the instrument’s line of sight [10].

This can be ameliorated with the use of fibre optic infrared radiation thermometers (FO-IRTs) by positioning the fibre tip close to the area of interest to collect and transmit the infrared light radiating from the target object to the photodiode sensor. FO-IRTs enable infrared temperature measurements by providing a better line of sight and protecting the electronics from otherwise deleterious environments [10,11]. 

The inherent challenge facing FO-IRT configurations is the low levels of infrared radiation emitted by objects at near-ambient temperatures, resulting in a low signal-to-noise ratio, and this is exacerbated at higher measurement speeds. Furthermore, traditional IRT configurations make use of silica fibres because they are relatively inexpensive and have relatively high bend radii. However, the use of such fibres limits the instrument’s cut-off wavelength to approximately 2.4 μm, which limits the lowest measurable temperature of the instrument.

Fibres with a wider transmission range, from mid-wave infrared (MWIR) between 3 and 5 μm to long-wave infrared (LWIR) between 8 and 14 μm [12,13], including chalcogenide and silver halogenide fibres, can transmit the emitted radiation from targets at near-ambient temperatures. The use of such fibres in FO-IRT configurations can help overcome the limitations of traditional FO-IRT configurations [7].

Traditionally, the detectors used for MWIR and LWIR IRTs have been thermopiles and mercury cadmium tellurium (HgCdTe or MCT) detectors [14]. Thermopiles consist of multiple thermocouples placed in a suspended dielectric layer with an absorbent layer on top, which is why they are often referred to as infrared thermocouples. Consequently, the challenges of thermopiles are not dissimilar to those of thermocouples, with their main drawbacks being their relatively slow response time and temperature drift [15]. MCTs have a very high temperature coefficient and therefore require cooling with thermoelectric cooling or liquid nitrogen, which is undesirable for many industrial and scientific applications [16,17,18].

The use of indium arsenide antimony (InAsSb) MWIR and LWIR photodiode detectors for IRTs is an emerging method that has the potential to fulfil the demand for fast and reliable near-ambient temperature sensing for many scientific and industrial applications [10]. These detectors have cooled and uncooled variants, allowing for smaller footprint, lower cost, and less complicated circuitry [19,20]. Alternatives such as indium arsenide (InAs) and Type II superlattice detectors are available; however, they are undesirable because they are very expensive and require cryogenic cooling [21,22]. The industrial sectors that would benefit from high-speed, near-ambient temperature sensing include those with near-ambient thermal cyclic conditions, for instance, conventional machining of polymers and polymer composites, cryogenic machining, electric motor condition monitoring, and battery protection. 

In this paper, we present a fibre optic infrared radiation thermometer, sensitive to wavelengths between 3.0 μm and 11.0 μm, for high-speed, near-ambient temperature measurements. We compare the IRT’s noise performance and uncertainty to that of Class 1 thermocouples. Our comparison demonstrates the benefits and capabilities of our FO-IRT based on an InAsSb LWIR detector and a silver halogenide optical fibre for high-speed, near-ambient temperature measurements. Our instrument is capable of measuring a target temperature above 35 °C at an integration time of 5 ms and a target temperature above 40 °C at an integration time of 1 ms, with a noise performance of better than ±0.5 °C. To the best of our knowledge, this is the first instrument to use an InAsSb photodiode detector to measure near-ambient temperatures at high speeds. Lastly, the fibre of the IRT was embedded in a block of polyether ether ketone (PEEK) during drilling trials to demonstrate its capabilities and efficiency in monitoring the temperature of the drill bit.

## 2. Experimental Methods

### 2.1. Instrument Design

**Figure 1:**
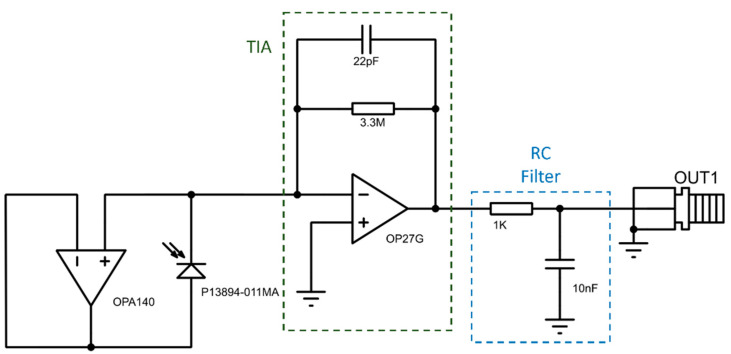
InAsSb photodiode transimpedance amplifier schematic.

The thermometer’s electronics comprised an uncooled, Hamamatsu P13894-011MA (Hamamatsu, Shizuoka, Japan), indium arsenide antimony (InAsSb) photovoltaic detector [23] and a transimpedance amplifier (TIA) circuit. The TIA comprised an OPA27G operational amplifier and a resistor-capacitor (RC) feedback network of 3.3 MΩ and 22 pF. This was followed by a first-order low-pass RC filter (1 kΩ and 10 nF) for further filtering, leading to an overall circuit time constant of approximately 83 μs, as depicted in the schematic in Figure 1. The photodiode had an active area of 1.0 mm by 1.0 mm and operated over a nominal wavelength range of 3.0 to 11.0 μm with a peak sensitivity at 5.6 μm [23]. The TIA was used to convert the photocurrent to a proportional output voltage that standard laboratory equipment is capable of measuring.

A polycrystalline silver halogenide (AgCl:AgB) optical fibre, by Art Photonics GmbH (Berlin, Germany), was used to transmit the infrared radiation from the measurand to the photodiode [24]. This allowed the sensing electronics to be protected through being positioned away from the harsh environment. The optical fibre used was 400 mm in length, with a core diameter of 860 μm, and had a transmittance in the wavelength region of 3–17 μm; optical losses of less than 0.3 dB/m; and numerical aperture of 0.3, equivalent to a conical half angle of acceptance of 17.45° [24].

### 2.2. Infrared Radiation Thermometer Characterisation

To characterise and assess the basic performance parameters, the electronic noise as a function of temperature, and the working lower temperature range of the IRT, a blackbody approximation furnace was used. An AMATEK LAND (Dronfield, UK) Landcal P80P blackbody reference furnace [25], with a manufacturer-quoted emissivity of greater than 0.995, was used. The thermometer’s performance was evaluated at source temperatures between 25 °C and 100 °C in 5 °C increments. 

**Figure 2:**
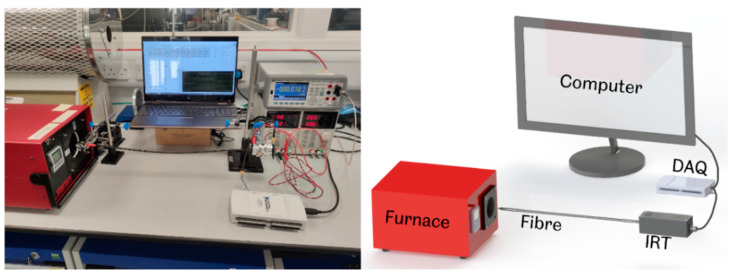
Configuration of the experimental apparatus used for the IRT characterisation.

The IRT was directly coupled with the fibre using an SMA905 connector, transferring all the emitted radiation from the target onto the detector. The tip of the fibre was concentrically and colinearly positioned directly in front of the furnace cavity, as depicted in Figure 2. The output of the IRT was connected to a power supply and a National Instruments (Austin, TX, USA) NI USB-6212 data acquisition device (DAQ), which recorded and sent the output voltage of the TIA to a computer every 10 μs [26]. Before any measurements were acquired, the blackbody furnace was given sufficient time to reach equilibrium at the specified temperature set point. This approach enabled optimum source temperature uniformity to be achieved. A LabVIEW (version 20.0.1) program was used to record the acquired data and simulate the thermometer’s performance at various acquisition times. The program simulated the noise performance at instrument integration times of 400 μs, 1 ms, 5 ms, and 10 ms.

For each integration time and source temperature, the thermometer’s mean output voltage and its standard deviation were evaluated to determine the noise and the root mean squared (RMS) noise of the thermometer configuration. At the start of each set of measurements, the instrument was blanked to acquire data at ambient temperature. To achieve a “zero” offset, the mean output voltage recorded from the ambient measurement, also referred to as a “dark voltage”, was subtracted from every measurement point. The product of this subtraction was used as the output voltage at the corresponding source temperature. 

Planck’s law represents the relationship between the radiant emittance of a blackbody as a function of temperature and wavelength, as shown in Equation (1) [27].

(1) $$L\left(\lambda ,T\right)=\frac{c_{1}}{\lambda^{5}}exp\left(\frac{c_{2}}{\lambda T}\right)\;$$

*L* represents the spectral radiance of a perfect blackbody, whilst *c*_1_ and *c*_2_ represent the first and second radiation constants, respectively. The temperature and wavelength are represented by *λ* and *T*, respectively. The photocurrent generated by the photodiode, *I_ph_*, is proportional to *L* and, subsequently, so is the IRT’s output voltage, *V*.

To characterise the IR thermometer, the linearity of the output voltage in the logarithmic scale (as a function of source temperature, as described by Wien’s approximation in Equation (2)) was assessed.

(2) $$V=exp\left(\frac{1}{T}m-c\right)$$

The relationship between the inverse absolute source temperature 1T and the natural logarithm of the IRT’s mean output voltage $\left(\ln{(V}_{o})\right)$ is used to aid in the assessment of the IRT’s linearity [27]. From this relationship, the mean effective wavelength, which is used to characterise the “operating wavelength” of IRT, can be calculated [28,29]. This allows for a direct comparison between different IRTs. 

**Figure 3:**
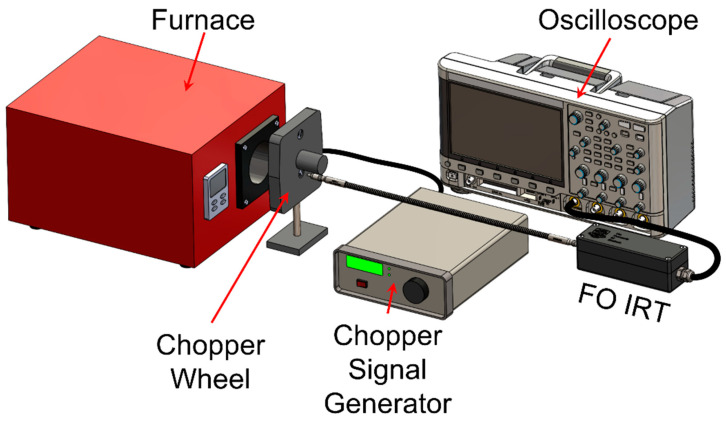
Response time measurement apparatus configuration.

The response time of the IRT is represented by the instrument’s rise time. This is the time required for the device output to rise from 10% to 90% of the steady-state value when subjected to a step-change of the input signal. The thermocouple response time is indicated by the thermocouple time constant, τ, which is the time required to display a sudden change in signal to 63.2%. To evaluate the IRT’s response performance, the blackbody approximation furnace was used as a heat source with a Terahertz Technologies Inc. (Oriskany, NY, USA) C-995 optical chopper positioned between the tip of the fibre and the furnace cavity, as shown in Figure 3 [30]. The optical chopper wheel was set to rotate at a frequency of 500 Hz, interrupting the line of sight between the fibre tip and the furnace cavity. The IRT signal output was captured using an oscilloscope with a temporal resolution of 1 μs. 

Calibration of the IRT was carried out using Equation (1), incorporating the wavelength-dependant responsivity of the photodiode and the spectral transmission of the fibre to produce the “shape” of the IRT output as a function of temperature. Using the output voltage corresponding to five calibration recorded temperatures, the calibration model was scaled to create a final calibration lookup table mapping output voltage, *V*, to temperature, *T*.

**Figure 4:**
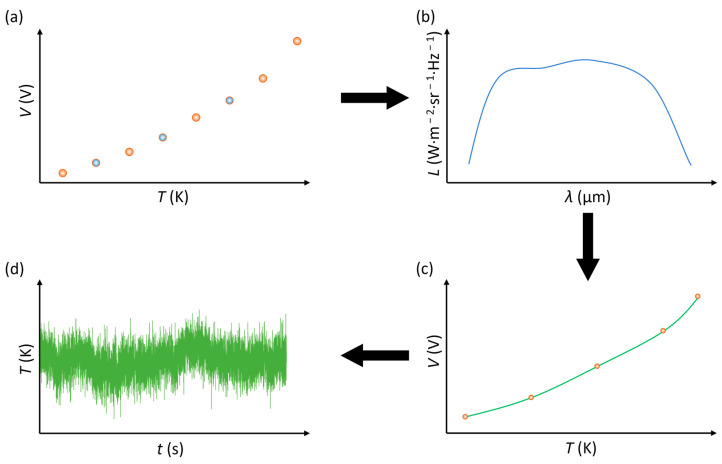
Infrared radiation thermometry calibration steps (**a**–**d**) (based on Hobbs et al. [31]).

A simplified calibration procedure for the infrared radiation thermometer is shown in Figure 4. Firstly, the output voltage as a function of target temperature was measured, with the dark voltage measured and subtracted from each point, as shown in Figure 4a. Secondly, the shape of the spectral radiance, *L*, against wavelength, *λ*, was obtained via modelling Planck’s law with the detector responsivity, and the fibre’s spectral transmission was plotted, as shown in Figure 4b. Using the five calibration points selected, the calibration model was scaled to create a calibration lookup table mapping the output voltage to temperature to obtain a calibration curve, as shown in Figure 4c. Lastly, the calibration was applied to the voltage output of the IRT to produce a temperature-over-time plot, as shown in Figure 4d.

### 2.3. Embedded Thermocouple

**Figure 5:**
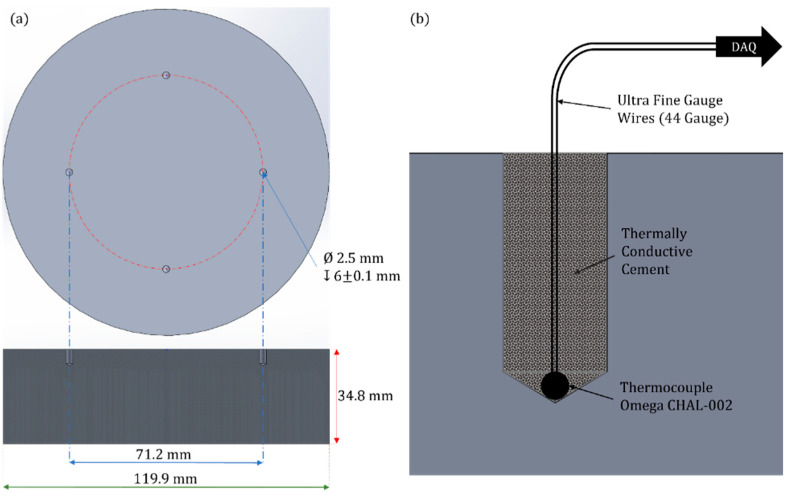
(**a**) Aluminium disc with blind holes and dimensions; (**b**) schematic of the embedded thermocouples inside the blind holes.

To evaluate the thermocouple’s repeatability and reliability, three type-K thermocouples (OMEGA CHAL-002) with a maximum service temperature of 593 °C and a manufacturer-rated response time of 5 ms [32,33] were embedded inside blind holes. The holes were drilled to a diameter of 2.5 mm and a depth of 6 mm on an aluminium disc, as shown in Figure 5. The holes were filled with high thermal conductivity cement, Omega Bond OB-400 (Omega Engineering, Manchester, UK), with a thermal conductivity of 1.59 W/m-K [34], to allow for good thermal contact with the aluminium disc.

The embedded thermocouples were connected to National Instruments DAQ hardware, consisting of a 9213 NI Module [35] and a 9171 Compact DAQ (cDAQ) chassis [36]. NI hardware was used to collect the thermocouple data, with a sample rate of 100 Hz, as the aluminium disc was heated in an ambient-temperature oven (Binder KT053-230V) [37] to evaluate the noise performance of the thermocouples. Thermocouple measurements were taken at source set points from 30 °C to 100 °C in 5 °C increments. For each measurement, sufficient time was allowed to ensure that the aluminium disc was in thermal equilibrium. The temperature fluctuation for each thermocouple was recorded and averaged to compare it with the FO-IRT.

## 3. Results and Discussion

### 3.1. Comparison of the Noise Performance of the IRT and the Thermocouple

**Figure 6:**
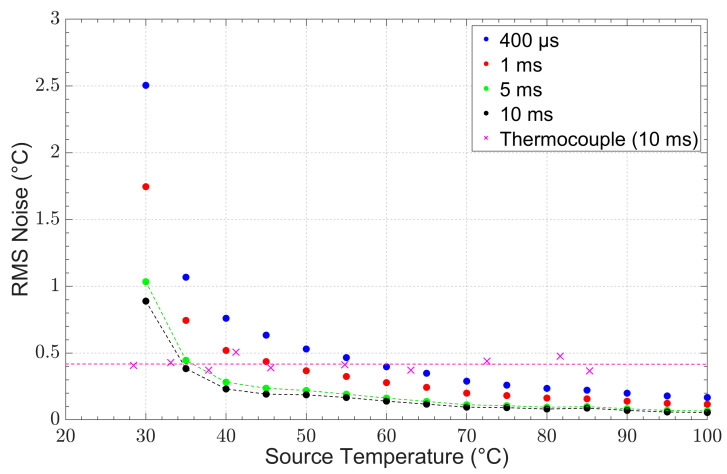
RMS noise as a function of target temperature for integration times of 400 μs, 1 ms, 5 ms, and 10 ms for the IRT and 10 ms for the thermocouple.

The root mean square (RMS) noise of the thermometer (at integration times of 400 μs, 1 ms, 5 ms, and 10 ms) was evaluated at source temperatures between 30 °C and 100 °C. The noise performance of the IRT at different integration time was compared to the noise performance of the thermocouple, at an acquisition time of 10 ms, as presented in Figure 6. The RMS noise was defined as the temperature equivalent (in degrees Celsius) of the standard deviation of the signal. It can be observed that as the source temperature increased, the RMS noise improved for all integration times as the RMS noise decreased. Furthermore, the effect of the thermometer’s integration time on the RMS noise can also be observed. Measurements at faster integration times display more noise than those at slower integration times. 

We defined the minimum measurable temperature of the instrument at an RMS noise of ±0.5 °C, which, in our experience, is the typical noise specification of commercial radiation thermometers. The instrument’s minimum measurable temperature for integration times of 5 ms and 10 ms was defined at 35 °C, and, for an integration time of 1 ms or longer, the minimum measurable temperature was found to be 40 °C. The IRT was able to match the performance of the thermocouple at 35 °C with an integration time twice that was as fast as that of the thermocouple. At 40 °C, the IRT was capable of matching the thermocouple noise performance at an integration time that was 10 times faster than that of the thermocouple. 

**Figure 7:**
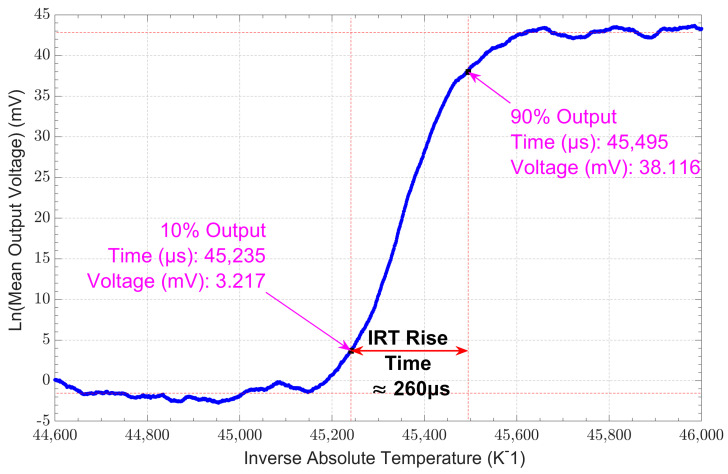
Rise time assessment of the IRT with an optical chopper at a frequency of 500 Hz at a target temperature of 80 °C.

When evaluating the IRT’s response to a sudden change in signal, its rise time was determined to be 260 μs, as shown in Figure 7, which is faster than the IRT’s integration time of 1 ms. The thermocouple was rated by the manufacturer as having a response time of 5 ms, which is among the fastest commercially available thermocouples. The IRT responded 19 times faster than the thermocouple, enabling it to reliably capture a sudden change in temperature. 

Thermocouples achieving higher response times would be expected to coincide with a trade-off in their accuracy, with a change in tolerance from 1.5 °C for Class 1 to 2.5 °C for Class 2, as specified in BS EN 6058-2:1993 [38]. In addition, for thermocouples to achieve a faster response time, they require the use of smaller diameter wires to reduce the junction mass for a lower thermal inertia [39]. This reduction in size makes them incredibly fragile and also makes it hard to ensure adequate contact with the target area, especially when inserted inside a blind hole. 

This is not a limitation when using fibre optics because they do not require intimate contact and the output of the IRT would be consistently reliable if there are no apparent changes in emissivity. 

### 3.2. Thermometer Output Voltage Characterisation

**Figure 8:**
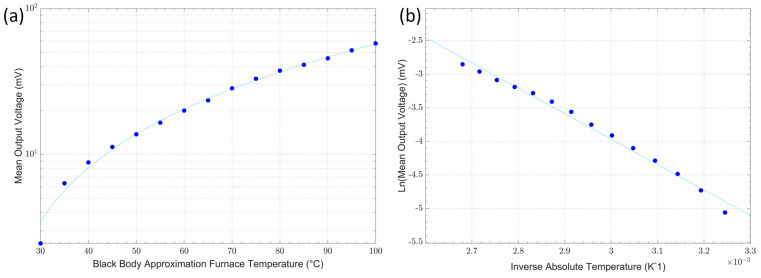
(**a**) Output voltage as a function of target temperature; (**b**) characteristic inverse absolute temperature against the natural logarithm of the output voltage.

The thermometer’s mean output voltage as a function of the blackbody approximation furnace (source) temperature between 30 °C and 100 °C is graphically presented on a logarithmic linear scale in Figure 8a.

The linearity of the thermometer configuration was evaluated from the plot of the inverse source temperature against the natural logarithm of the mean output voltage that should be expected from differentiating Wien’s approximation. The characteristic curve measured for this thermometer is graphically presented in Figure 8b. There is a small deviation from the linear relationship at the lower end of the temperatures measured owing to the signal level, which is approaching the instrument noise floor. Based on the gradient of the curves plotted in Figure 8b, the mean effective wavelength was estimated to be 3.97 μm [28,29,40].

### 3.3. Thermometer Calibration and Error Analysis

**Figure 9:**
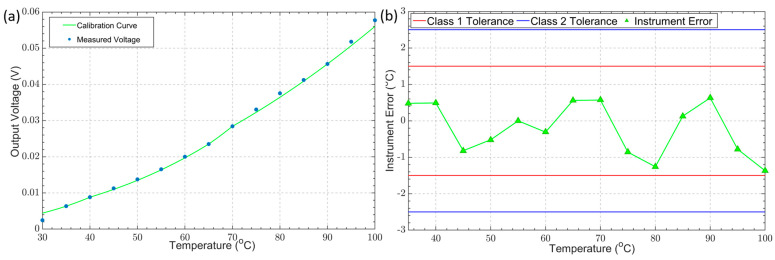
(**a**) Calculated and measured output voltages against temperature (calibration); (**b**) instrument error after calibration compared to the thermocouple tolerance bounds for Class 1 and Class 2 tolerances.

Using the model of Planck’s law, the expected “shape” of the output voltage at different source temperatures was calculated. Following a five-point calibration at temperatures of 35 °C, 40 °C, 65 °C, 70 °C, and 90 °C, the calibration curve was obtained. The graph of Figure 9a shows the calibration curve based on our model of Planck’s law after a five-point calibration and the measured output voltage of the IRT. The calibration curve shows good agreement with the IRT output voltage (R^2^ = 0.99896); however, there are some small deviations that are within the accepted range of the instrument’s uncertainty error. The uncertainty was quantified using the difference between the target temperature and the calculated temperature. Following a five-point calibration, the IRT’s uncertainty error was found to be within ±1.5 °C (or ±0.4%K). The IRT’s uncertainty error falls within the tolerance bounds for Class 1 (±1.5 °C) and Class 2 (±2.5 °C) thermocouples, as specified in the BS EN 60584-2:1993 standards for thermocouple tolerance (shown in Figure 9b) [38]. The instrument’s uncertainty error is also comparable with that of commercially available infrared radiation thermometers [41,42].

**Figure 10:**
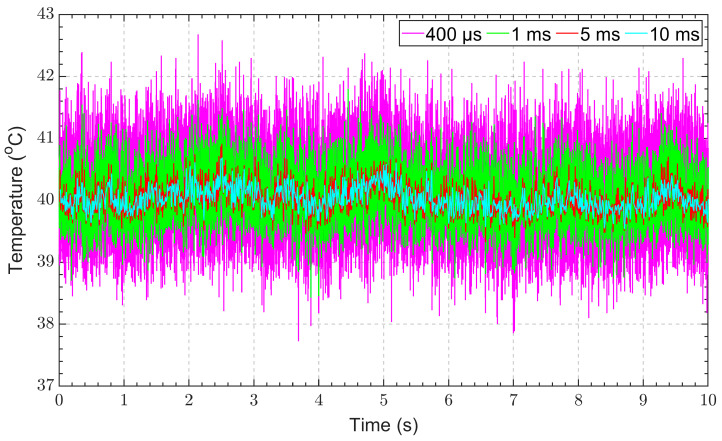
IRT temperature against time for acquisition times of 400 μs, 1 ms, 5 ms, and 10 ms for a target temperature.

Following the five-point calibration, the measured temperature of a blackbody target for integration times of 400 μs, 1 ms, 5 ms, and 10 ms was plotted over a 10 s measurement period. Figure 10 shows the calibrated measurements of a blackbody furnace at 40 °C. The measurement noise significantly decreased with longer integration times, with an RMS noise level of 0.76 °C, 0.52 °C, 0.28 °C, and 0.23 °C at integration times of 400 μs, 1 ms, 5 ms, and 10 ms, respectively.

### 3.4. Measurement Uncertainty Estimate

The uncertainty budget for the measurements acquired using the FO-IRT was estimated based upon the individual contributions from the equipment used in calibration and characterisation and the measurements acquired using the FO-IRT. The estimated contributions considered in the uncertainty budget estimate arising from the equipment and from the measurement are presented in Table 1 and Table 2, respectively.

The overall uncertainty was found to be 2.3% for a source temperature of 70 °C. The primary sources of uncertainty were identified as the variability in the FO-IRT measurements and the error associated with the interpolation during the conversion between voltage and temperature. The uncertainties that ensued from the calibration equipment can be neglected without detriment to the uncertainty estimate. Therefore, the uncertainty estimation of the FO-IRT can be simplified by only evaluating the uncertainty from the measurement variability and the interpolation error and reporting the root sum square of the individual contributions as the uncertainty of the FO-IRT.

### 3.5. Example of Implementation in Application

**Figure 11:**
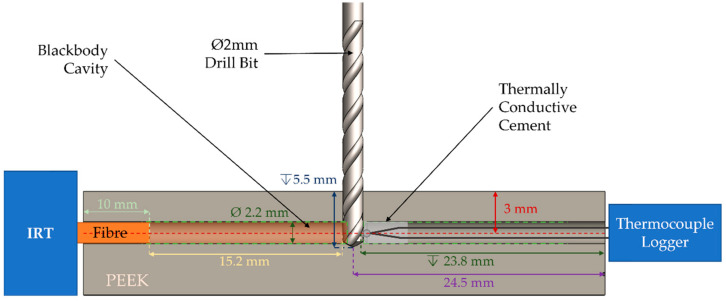
Arrangement of the IRT and thermocouple inside the PEEK workpiece during drilling operations on a manual milling machine.

The open end of the fibre of the IRT was embedded in a block of polyether ether ketone (PEEK) plastic, an example material, during drilling trials as an example of the implementation in application. The fibre was embedded at a depth of 10 mm through a 2.2 mm hole at the side of the workpiece and at a distance of 3 mm from the drilling surface. The arrangement of the fibre inside the cavity ensured that the apparent emissivity was at least 0.995, allowing for the cavity depth to be more than six times longer than the diameter of the fibre [43]. A thermocouple was positioned at a depth of 23.8 mm, inside an identical hole, at the same height on the opposite side of the workpiece, and the hole was filled with thermally conductive cement. Figure 11 depicts the arrangement of the fibre and the thermocouple inside the PEEK workpiece. The IRT and the thermocouple outputs were synchronously recorded using the same configuration as mentioned in Section 2. A 2 mm drill tool was used to form a 5.5 mm deep, blind hole on the top face of the PEEK material, using a manual milling machine.

**Figure 12:**
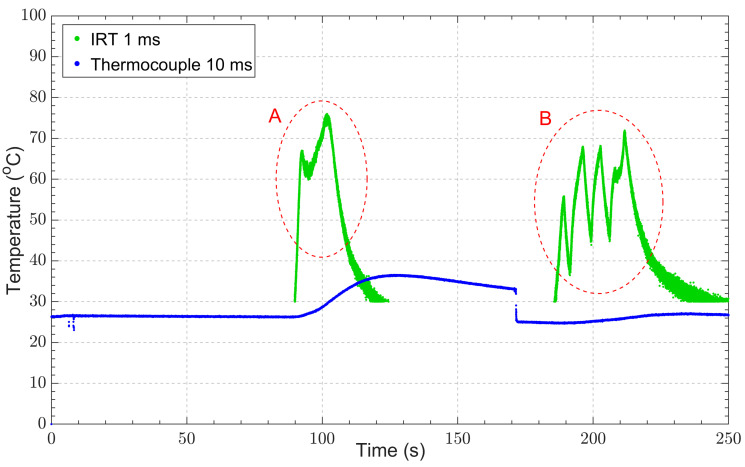
IRT and thermocouple temperature measurements during drilling operations.

The measured temperatures from both the thermocouple and IRT during the drilling operation are shown in Figure 12. The IRT measurements were taken at an integration time of 1 ms, and the thermocouple readings were taken every 10 ms. The peak temperature measured using the IRT, at 75.97 °C, were within the expected range and similar to temperatures reported in the literature for similar drilling operations of PEEK recorded with thermal cameras observing the drilling surface [44,45].

The events in the circled region A in Figure 12 show the temperature rise during the material removal process, drilling to a depth of 5.5 mm. The IRT measurements show a sharp rise and a sharp decline in temperature during this initial material removal process. The IRT was able to capture the sharp rise in the drill bit temperature, whereas the thermocouple suffered from thermal lag resulting from the thermocouple junction not being in direct contact with the drill bit. In contrast, the thermocouple measurements show a slow increase in temperature as well as a very slow decrease in temperature due to the low thermal conductivity of plastics such as PEEK. 

The events in the circled region B in Figure 12 show the drill bit re-entering the hole without cutting, to a depth of 5.5 mm, and fully exiting the hole four consecutive times, which indicated the drill bit was still at a peak temperature of 70 °C. These events were clearly captured by the IRT but not by the thermocouple. The non-contact nature of the IRT allowed these events to be captured, whereas the thermocouple required reliable direct contact, which was not feasible during these events.

The limitations of thermocouples for high-speed, near-ambient temperature measurements can easily be observed from the drilling experiments. Their positioning plays an important role in their sensitivity, as does the reliability with which the temperature at the area of interest can be measured [46]. Perhaps the most significant demonstration of the thermocouple’s limitation is the delayed and slow response to temperature change. The thermocouple’s performance could be improved through better positioning of the thermocouple junction; however, this is extremely difficult to achieve and increases the risk of irreversible damage to the thermocouple, which is one of the main drivers of the use of non-contact temperature measurement techniques.

In contrast, our FO-IRT demonstrated that it can overcome thermocouple limitations, rapidly capturing sudden changes in temperature. The peak temperature readings were similar to those reported in the literature, corroborating our FO-IRT measurements. 

## 4. Conclusions and Further Implementations

In this paper, a novel uncooled InAsSb photodiode fibre optic thermometer was presented for high-speed, near-ambient temperature measurements, and its performance was compared to that of high-speed thermocouples. The minimum temperature of the instrument was defined at an RMS noise resolution of ±0.5 °C, which was achieved at 35 °C and 40 °C for integration times of 5 ms and 1 ms, respectively. To the best of our knowledge, this to be the fastest FO-IRT to utilise an uncooled LWIR detector for temperature measurements between 35 °C and 100 °C. The instrument’s response time was found to be 260 μs, which is more than 19 times faster than that of the fastest commercially available thermocouples. It was determined that after calibration, the IRT’s accuracy was within the tolerance bounds of a Class 1 thermocouple, with an instrument uncertainty error of less than ±1.5 °C and an order-of-magnitude faster measurement. 

The instrument was demonstrated to be a suitable thermocouple replacement for industrial and scientific use by performing temperature measurements during drilling operations with PEEK. The FO-IRT configuration was able to record a peak temperature of 76.0 °C during the material removal process and capture the sudden changes in temperature during the operation, which is not possible with contemporaneous thermocouple measurements. The same implementation could be employed to obtain reliable high-speed temperature measurements for the machining of composite materials, such as carbon-fibre-reinforced polymer (CFRP). 

It is thought that this technology has great potential for integration into industrial processes for real-time temperature monitoring in many scientific and industrial sectors subject to high-frequency thermal cyclic events. These sectors include conventional machining, cryogenic machining, using tool- and workpiece-embedded techniques where the temperature is within the operating range of the FO-IRT, electric motor condition monitoring, and battery protection. Our findings contribute to the development of advanced temperature measurement techniques and pave the way for further research in this field.

## Figures and Tables

**Table 1:** Contributions of calibration uncertainties to overall measurement uncertainty.

Source Temperature (°C)	Calibration Thermocouple Uncertainty (%)	Calibration Blackbody Uncertainty (%)	DAQ Uncertainty (%)
70	0.4	0.3	0.0015

**Table 2:** Contributions of measurement uncertainties to overall measurement uncertainty.

Source Temperature (°C)	Infrared Thermometer Variability in Usage (%)	Interpolation Error of Thermometer Mean Measurement (%)
70	1.1	2.0

## Data Availability

Data is contained within the article.
